# Bio-Based Rigid Polyurethane Foams Modified with Phosphorus Flame Retardants

**DOI:** 10.3390/polym14010102

**Published:** 2021-12-28

**Authors:** Marcin Zemła, Aleksander Prociak, Sławomir Michałowski

**Affiliations:** Faculty of Chemical Engineering and Technology, Cracow University of Technology, Warszawska 24, 31-155 Krakow, Poland; slawomir.michalowski@pk.edu.pl

**Keywords:** rigid polyurethane foam, phosphorus flame retardant, bio-polyol, flammability

## Abstract

Rigid polyurethane foams (RPURF) containing a bio-polyol from rapeseed oil and different phosphorus-based flame retardants were obtained. Triethyl phosphate (TEP), dimethyl propane phosphonate (DMPP) and cyclic phosphonates Addforce CT 901 (20 parts per hundred polyol by weight) were used in the synthesis of RPURF. The influence of used flame retardants on foaming process, cell structure, and physical–mechanical properties as well as flammability of RPURF were examined. The addition of flame retardants influenced the parameters of the cellular structure and decreased compressive strength. All obtained foam materials had a low thermal conductivity coefficient, which allows them to be used as thermal insulation. The research results of bio-based RPURF were compared with foams obtained without bio-polyol. All modified materials had an oxygen index above 21 vol%; therefore, they can be classified as self-extinguishing materials. The analysis of parameters obtained after the cone calorimeter test showed that the modified RPURF have a lower tendency to fire development compared to the reference foams, which was particularly noticeable for the materials with the addition of DMPP.

## 1. Introduction

Rigid polyurethane foams (RPURF) are among the best thermal insulation materials produced on a large scale. By selecting the appropriate raw materials for the synthesis of polyurethane (PUR) foams, it is possible to obtain products with a wide range of properties [1]. These features, combined with good mechanical properties, make RPURF applicable in many branches of industry such as construction, automotive, transport, mainly as thermal insulations [2,3,4,5].

Due to their organic nature and well-developed specific surface, RPURFs are flammable materials, which is their major disadvantage [6]. Nowadays, there is an urgent need to modify PUR systems to increase fire resistance of produced foams [7,8]. Modifications should reduce all fire hazards, such as total heat release, smoke generation, the toxicity of gaseous decomposition products, and the rate of combustion. The application of proper flame retardants should allow for the safe evacuation of people, as well as reducing material losses caused by the fire [9]. Currently, the most common method of reducing the flammability of PUR materials is the addition of additive flame retardants during synthesis [2,10]. Organic nitrogen compounds, organic phosphorus compounds such as phosphates, phosphonates, phosphites, and others are widely used flame retardants [11,12,13]. These compounds can act effectively in the gas phase as radical scavengers and in the condensed phase, creating a charred layer on the polymer surface, which reduces the flow of heat and gas products of thermal decomposition of the material [4,14,15]. The condensed-phase flame retardant action reduces the emission of smoke and toxic combustion products. In the gas phase, the radicals resulting from the decomposition of the flame retardant recombine with the hydrogen (H^•^) and hydroxide (OH^•^) radicals, stopping the oxidation reactions [16]. Expanded graphite [9,17], aluminum hydroxide [18], magnesium hydroxide [19], ammonium polyphosphate [20], as well as layered clay [21] are also frequently used substances. However, to achieve an acceptable reduction in the flammability of PUR, inorganic substances often have to be used in large amounts, which adversely affect the structure of the material and the physical and mechanical properties [22]. Effective flame retardancy is also ensured by compounds containing chlorine or bromine in their structure. However, the use of these compounds is currently limited due to their toxicity and negative environmental impact [18,23,24].

Due to the growing interest in PUR plastics and the sustainable development policy, solutions are sought to replace polyols of petrochemical origin with polyols obtained from renewable raw materials [25,26]. Bio-polyols can be obtained from various types of vegetable oils, e.g., rapeseed [27,28], soybean [29], sunflower, corn [26], palm [30], tall [31], and castor [32] oils. Most vegetable oils do not contain functional groups in their chemical structure capable of reacting with isocyanate groups; therefore, it is necessary to modify them by introducing hydroxyl groups [30]. Because vegetable oils are esters of fatty acids and glycerin, the synthesis of bio-polyols may occur as a result of transesterification and transamidization [33]. Another way is to modify the unsaturated fatty acid bond, i.a., by hydroformylation with synthesis gas, and then reduction in aldehyde groups with hydrogen, ozonolysis, or epoxidation of the double bonds and opening of the reactive oxirane ring [31,33,34]. The use of plant-derived polyols allows for the reduction in the carbon footprint and is seen as an appropriate solution in the circular economy model, making the production of PUR materials more environmentally friendly [31].

The aim of this research was to obtain RPURF containing new bio-polyol from rapeseed oil synthesized in our laboratory [27] and various liquid phosphorous flame retardants, and analyze their effect on useful properties as potential thermal-insulating materials. Therefore, the influence of the applied modifiers on the foaming process was determined, along with selected physical and mechanical properties and the flammability of foamed PUR materials. The test results were compared for RPURF modified with the same flame retardants but obtained with and without bio-polyol based on rapeseed oil.

## 2. Materials and Methods

### 2.1. Materials

Petrochemical polyether polyol Rokopol^®^ RF-551 with a hydroxyl number of 420 mgKOH/g and viscosity of 4000 mPa·s was provided by PCC Rokita S.A, Brzeg Dolny. Bio-polyol 1.6Hex with a hydroxyl number of 217 mgKOH/g, water content of 0.25 wt.% and viscosity of 2050 mPa·s was synthesized in the Department of Chemistry and Technology of Polymers at the Cracow University of Technology [27]. Bio-polyol was obtained by epoxidation of double bonds in rapeseed oil and then opening of the oxirane rings used 1,6-hexanediol. Polymeric methylene diphenyldiisocyanate EKOPUR B (PMDI) with an isocyanate group content of 31 wt.% was supplied by Minova Ekochem S.A. Catalyst Polycat^®^ 218, reactive amine catalyst was supplied by Evonik Industries AG. Surfactant Niax^®^ Silicone L-6915 was produced by Momentive Performance Materials Inc. Water was used as a chemical blowing agent. The following flame retardant were used: triethyl phosphate (TEP) with a phosphorus content of 17 wt.% and viscosity of 1.7 mPa·s supplied by Purinova, dimethyl propane phosphonate (DMPP)-Levagard^®^ DMPP with a phosphorus content of 20.3 wt.% and viscosity of 2.5 mPa·s provided by Lanxess and Addforce CT 901, cyclic phosphonates (CT901) with a phosphorus content of 19 wt.%, viscosity of 1500 mPa·s and water content of 8.2 wt.%, supplied by WTH Walter Thieme Handel GmbH.

### 2.2. Preparation of Polyurethane Foams

RPURFs were obtained by mixing a polyol premix with surfactant, catalyst, water, and flame retardants (component A) and an isocyanate (component B) for 5 s with a mechanical stirrer. The mixture was poured into an open mold, which allowed the foam to free-rise in a vertical direction and then to cross-link. After synthesis, the foam was seasoned for 24 h at room temperature. Table 1 shows the two reference PUR systems and their modifications with the flame retardants. One of PUR systems contained a petrochemical polyol (P_REF). The second system was made of a mixture of 60 wt.% of petrochemical polyol and 40 wt.% of bio-polyol (1.6Hex_REF). The reference systems were modified with the addition of liquid additive flame retardants (TEP, DMPP, and CT901) in the amount of 20 wt.% relative to the weight of the polyols components. In the calculation of total water content in the PUR systems, the water content in the Addforce CT 901 and the bio-polyol was also taken into account.

### 2.3. Test Method

The foaming process was analyzed using FOAMAT^®^ equipment. The analysis was based on the measurement of changes in the dielectric polarization of the foamed mixture, as well as the temperature during the foaming process, which allows determining the reactivity of the system.

During the synthesis, characteristic foaming times were measured with an electronic stopwatch, such as gelation time (from the beginning of mixing the components A and B until it is possible to pull the thread out of the foam), rise time (from the beginning of mixing the components A and B until the foam stop rise), and tack-free time (from the beginning of mixing the components A and B until the foam surface does not stick to the glass rod).

The apparent density of foams was examined according to ISO 845. The closed-cell content was measured according to ISO 4590. Water absorption was measured according to ISO 2896. The thermal conductivity coefficient of the obtained foams was measured using a Laser Comp Heat Flow Instrument Fox 200 at an average temperature of 10 °C according to ISO 8301.

Microphotographs of the cellular structures were taken using an optical microscope equipped with a camera. The obtained photos were analyzed with the Aphelion software, calculating the number of cells per 1 mm^2^, the mean cell area, and the cell anisotropy index.

The compressive strength was measured in a direction parallel and perpendicular to the foam rise direction using cylindrical samples with a diameter and height of 40 mm according to ISO 844.

The dimensional stability test during seasoning at a specific temperature was analyzed based on the ISO 2796-1986. The test specimens had the shape of a cuboid with dimensions of 100 × 100 × 25 mm^3^. Changes in the linear dimensions of the samples were determined after 24 h seasoning at −30 °C, as well as at 70 °C and 90% humidity.

The brittleness of PUR foams was determined by the percentage weight loss of 12 cubic cubes with an edge length of 25 mm after turning them with 24 oak cubes with an edge length of 20 mm in an oak box for 10 min and a speed of 60 rpm according to ASTM C-421.

The combustion properties were tested by pyrolysis combustion flow calorimeter (PCFC) developed by Fire Testing Technology Ltd. according to the ASTM D7309 at a synthetic air mixture N_2_:O_2_ volume ratio 80:20 at a constant rate of temperature rise of 1 °C/min. The mass of the samples was ca. 2 mg. Measurements allow to obtain information on the total heat released (THR), the rate of heat release (HRR), the heat release capacity (HRC) as well as the temperature of the thermal decomposition stages of the material. The limiting oxygen index test was performed following ISO 4589-2.

Foams were also tested with a FTT cone calorimeter (Fire Testing Technology Ltd., East Grinstead, UK) according to ISO 5660-1 at an external heat flux of 35 kW/m^2^ for 300 s. Separation space between samples and the heater was set at 25 mm. The time to ignition (TTI), total heat release (THR), peak heat release rate (PHRR), average heat release rate (Av-HRR), maximum average rate of heat emission (MARHE), average effective heat of combustion (Av-EHC), total smoke release (TSR), total smoke production (TSP), average yield of CO (Av-COY), average yield of CO_2_ (Av-CO_2_Y), and char residue were determined.

## 3. Results and Discussion

### 3.1. Foaming Process

**Figure 1:**
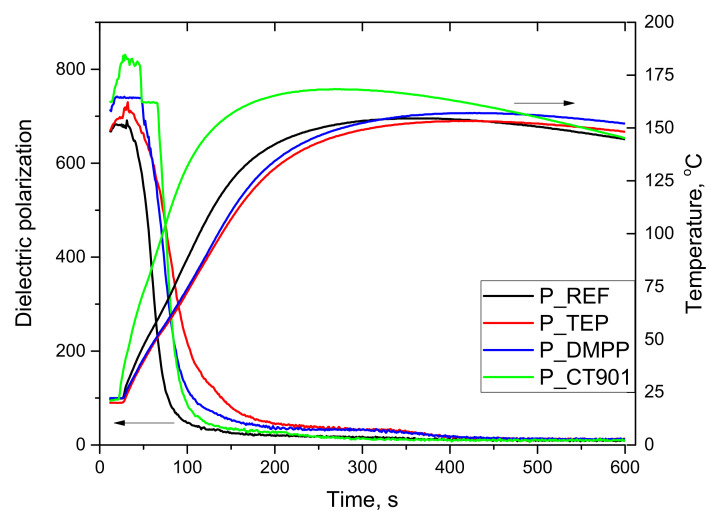
Influence of flame retardants addition to the reference system on dielectric polarization and temperature during the PUR foaming process without the addition of bio-polyol.

**Figure 2:**
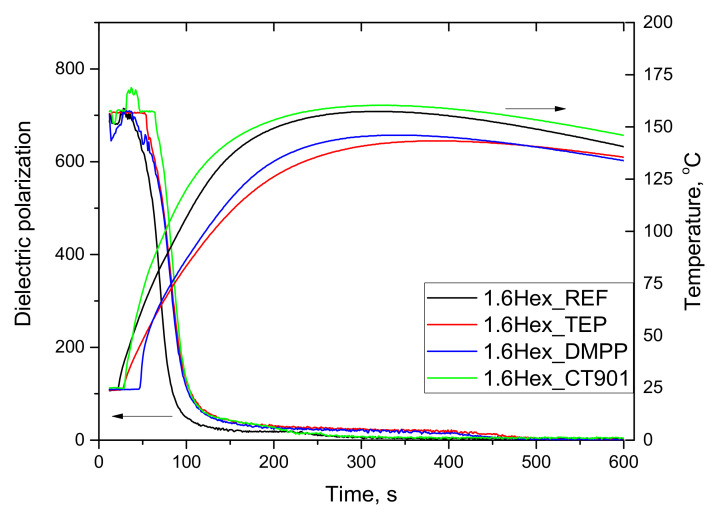
Influence of flame retardants addition to the reference system containing the bio-polyol on dielectric polarization and temperature during the foaming process.

The foaming process of tested PUR systems was analyzed using a FOAMAT^®^ device. The changes in dielectric polarization and temperature during the foaming process of the reference system using petrochemical polyol (Figure 1) and the system modified with 40 wt.% of the bio-polyol (Figure 2) were determined. The decrease in dielectric polarization of reacting systems during foaming results from the reduction in mobility and then stopping the movement of the dipoles as a result of polyaddition reaction. The dependence of the dielectric polarization on the reaction time reflects the reactivity of the polyurethane system [35]. The addition of flame retardants reduced the reactivity of both used PUR systems. However, in the case of P_CT901 system, the maximum temperature during foaming was higher than in the case of the reference material. This explains the faster change in dielectric polarization from about 80 s of reaction compared to other modified materials. The maximum P_TEP and P_DMPP temperatures were close to P_REF.

The system modified with the bio-polyol and Addforce CT 901 was also characterized by the highest temperature. In this system, as in the case of P_CT901 system, the dielectric polarization changed faster in the second stage of reaction compared to other modified PUR 1.6Hex_TEP and 1.6Hex_DMPP materials reaching a lower maximum temperature during the foaming process, equal to 143 °C and 146 °C, respectively, compared to 1.6Hex_REF (157 °C).

**Figure 3:**
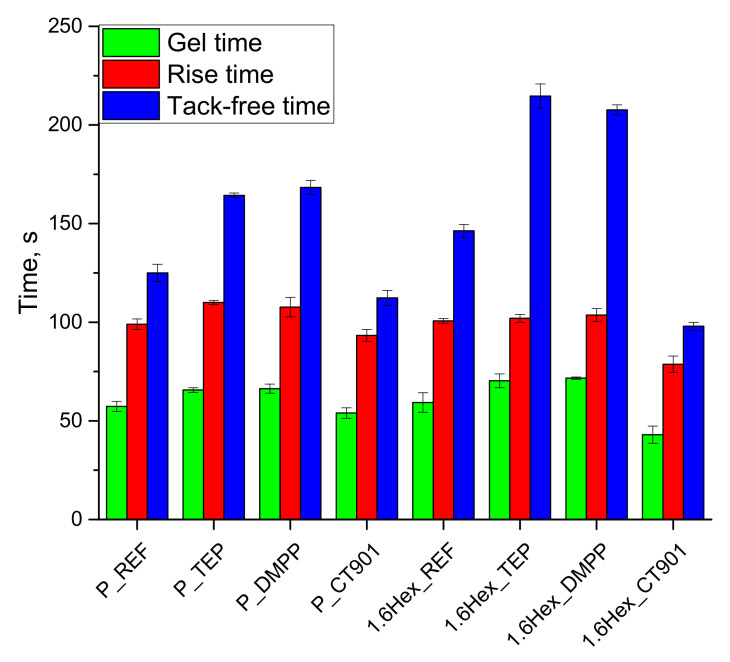
Characteristic processing times during the synthesis of PUR foams.

During the synthesis of RPURF, appropriate processing times were measured, such as rise time, gel time, and tack-free time (Figure 3). Both, unmodified systems based on the petrochemical polyol only (P_REF) and on a mixture of the petrochemical polyol and the bio-polyol (1.6Hex_REF) had similar gel and rise times, but the tack-free time for 1.6Hex_REF was longer by 20 s compared to P_REF. The addition of TEP and DMPP caused the extension of all processing times. In the case of P_TEP and P_DMPP foams, the rise and gel times increased by 10 s and the tack-free time by 40 s compared to the reference material based on petrochemical polyol. The addition of TEP and DMPP to the PUR system containing the bio-polyol did not significantly affect the rise time, but the gel time was extended by 11 s and 13 s, respectively. The tack-free time also increased from 146 s for 1.6Hex_REF foam to 215 s and 208 s for 1.6Hex_TEP and 1.6Hex_DMPP foams, respectively. The addition of CT 901 reduced all processing times in both type systems. The rise, gel, and tack-free times of the P_CT901 foam were shortened by 6 s, 3 s, and 13 s, respectively, compared to the P_REF foam. In the case of the system using the mixture of the petrochemical polyol and the bio-polyol with the addition of Addforce CT 901, these times were shortened by 22 s, 29 s, and 48 s, respectively, compared to 1.6Hex_REF. The change in processing times corresponds to the results of the foaming process analysis with the FOAMAT^®^ device. The shortening of the characteristic times after the addition of Addforce CT 901 may be caused by the higher initial viscosity of the foaming mixture compared to other systems, due to the higher viscosity of this flame retardant. The higher initial viscosity of the composition with Addforce CT 901 compared to other compositions, caused more heat to be generated during mixing, thus accelerating the reaction.

### 3.2. Cell Structure

**Figure 4:**
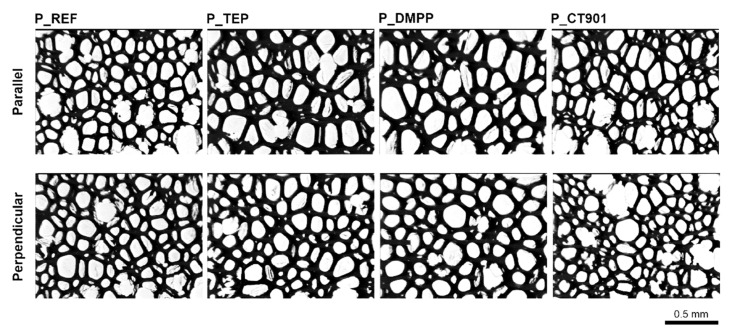
Microphotographs of the cellular structure of PUR foams without bio-polyol.

**Figure 5:**
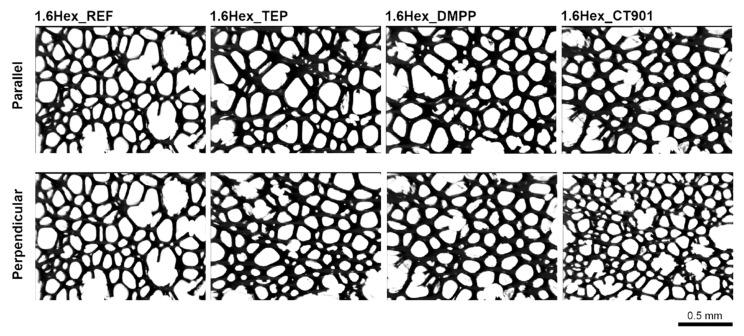
Microphotographs of the cell structure of polyurethane foams modified with 40 wt.% bio-polyol.

Due to the free rise of the obtained PUR foams, their properties are different depending on the test direction, as they are anisotropic materials. A number of properties are influenced by the shape and size of cells, which are elongated in the direction of foam rise (Figure 4 and Figure 5). For this reason, the study of the cellular structure should be performed in the direction parallel and perpendicular to the direction of foam rise. Table 2 and Table 3 show the parameters of the cellular structure of the obtained PUR foams. The addition of TEP and DMPP to the system with petrochemical polyol reduced the number of cells per 1 mm^2^ in the direction parallel to the foam rise. Modification of both systems (with and without the bio-polyol) with Addforce CT 901 caused that the number of cells per 1 mm^2^ increased and the average cell cross-sectional area decreased compared to the reference materials without flame retardants. In the case of PUR foams based on a mixture of petrochemical polyol and bio-polyol, the addition of TEP and DMMP did not cause significant changes in the number of cells and average cross-sectional area, but decreased the anisotropy index of cells evaluated in both cross-sections parallel and perpendicular to the foam rise direction. Materials synthesized with the use of bio-polyol were characterized by a higher number of cells with a smaller size compared to foams containing only petrochemical polyol. The used bio-polyol contains hydrophilic ester and hydroxyl groups as well as hydrophobic hydrocarbon chains; therefore, it can act as a surfactant, influencing the number and size of cells [27,36,37].

### 3.3. Physical and Mechanical Properties

RPURFs with the addition of TEP and DMPP based on both PUR systems had a higher thermal conductivity (Table 4) due to the lower content of closed cells and larger cells. In the case of the P_CT901 foam, despite the more favorable cellular structure, the value of the thermal conductivity coefficient was slightly higher than the reference material P_REF, and the value for 1.6Hex_CT901 was close to 1.6Hex_REF. This may be the result of moisture absorbed from the air by the hygroscopic flame retardant. Almost all foams had closed cells content above 90%. Only the 1.6Hex_TEP and 1.6Hex_DMPP foams had a slightly lower value (89%), which could slightly increase the value of the thermal conductivity of these foams compared to the reference foam.

The materials obtained with the use of petrochemical polyol and modified with TEP as well as DMPP had slightly lower water absorption compared to the P_REF foam (Table 4). The P_CT901 foam was characterized by 1.2% higher water absorption than the reference foam. The addition of DMPP and Addforce CT 901 to the PUR system containing the bio-polyol also increased the water absorption compared to 1.6Hex_REF by 1.0% and 3.8%, respectively. The 1.6Hex_TEP foam was characterized by a water absorption similar to that of the respective reference foam. Higher water absorption of foams with Addforce CT 901 may be the result of good solubility of this flame retardant in water.

The results of the compressive strength test are shown in Table 4. The presence of TEP in P_TEP and 1.6Hex_TEP foams only slightly decreased the compressive strength in the parallel direction and slightly increased the strength perpendicular to the direction of foam rise compared to P_REF and 1.6Hex_REF foams, respectively. The foams with the addition of DMPP showed a reduced strength compared to the unmodified foams, regardless of the direction of the test. The reduction in the compressive strength after the addition of DMPP results from the plasticization effect of the PUR matrix and larger cells compared to the cells in the reference foams [26]. Materials synthesized with the use of 40 wt.% bio-polyol had lower compressive strength compared to the foams obtained using petrochemical polyol only. This is the result of a higher content of elastic segments derived from the bio-polyol and a lower hydroxyl number of the bio-polyol compared to the petrochemical polyol [26,38]. The P_CT901 foam had a higher compressive strength parallel to the rise direction, but in the perpendicular direction the strength decreased by 26 kPa compared to P_REF. The addition of Addforce CT 901 to the bio-polyol-containing PUR system resulted in a strength reduction of 23 kPa in the direction parallel to the foam rise direction compared to 1.6Hex_REF, and in the perpendicular direction the compressive strength was reduced to 88 kPa. All materials except 1.6Hex_CT901 were characterized by compressive strength above 130 kPa. The addition of flame retardants only slightly increased the brittleness of the RPURF for both type PUR systems. The foams obtained with the bio-polyol were characterized by a slightly lower brittleness compared to the foams obtained solely from petrochemical polyol. This may be the result of the plasticizing effect associated with the addition of a bio-polyol of plant oil origin.

**Figure 6:**
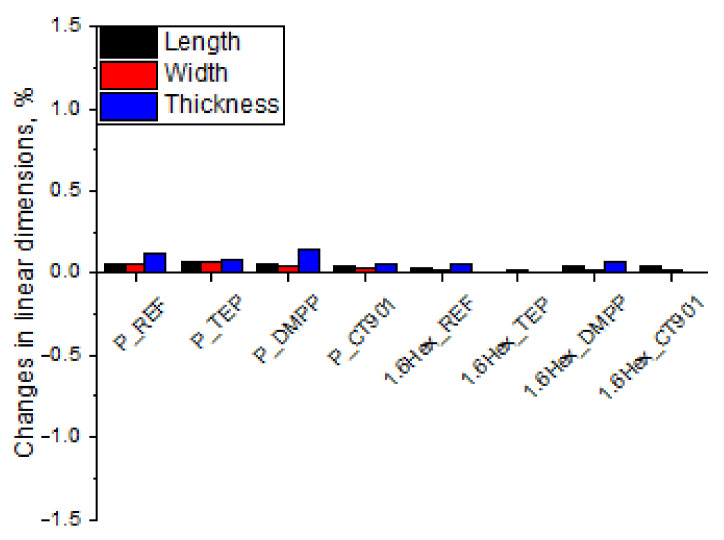
Changes in linear dimensions of RPURFs after seasoning at −25 °C for 24 h.

**Figure 7:**
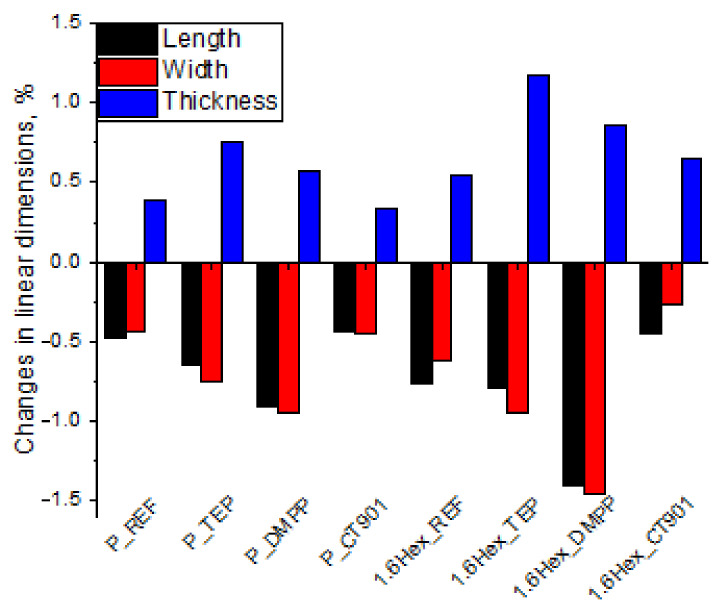
Changes in linear dimensions of RPURFs after seasoning at 70 °C and 90% humidity for 24 h.

The dimensional stability of RPURFs was determined from changes in linear dimensions parallel to the foam rise direction (thickness) and perpendicular to the foam rise direction (length, width) after seasoning at −25 °C as well as 70 °C and 90% humidity for 24 h. The study of dimensional stability at −25 °C (Figure 6) showed that the obtained materials are dimensionally stable at low temperatures. The change in linear dimensions never exceed 0.2%. After the foams were seasoned for 24 h at 70 °C and 90% humidity (Figure 7), the changes in dimensions were more significant. However, the change in linear dimensions of all materials was not bigger than 1.4%. The foams seasoned at 70 °C decreased their length and width, and increased their thickness.

### 3.4. Flammability Testing

**Figure 8:**
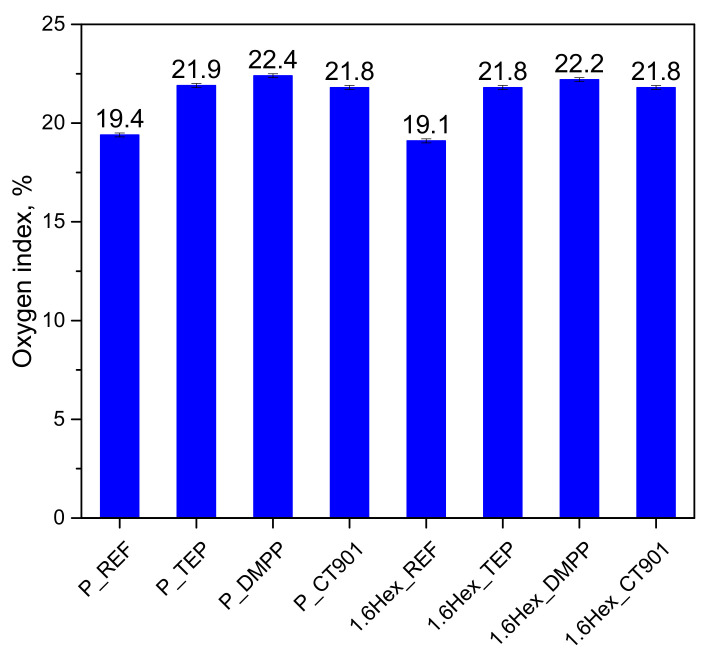
Limiting oxygen index of the obtained RPURFs.

The addition of flame retardants to PUR systems increased the oxygen index (OI) of the modified foams (Figure 8). The introduction of DMPP to both systems resulted in the highest increase in OI value compared to foams without the addition of flame retardants. The higher values of the oxygen index for the P_DMPP and 1.6Hex_DMPP foams were due to the fact that DMPP contains the highest amount of phosphorus among used flame retardants. The higher content of phosphorus in the modifier results in more effective creation of char and on foams surface and reduction their flammability. Materials containing TEP and Addforce CT 901 had a similar value of OI to about 21.8 vol%. Despite the lower OI value for 1.6Hex_REF compared to P_REF, materials modified with the same flame retardant have a similar OI value in both systems.

**Figure 9:**
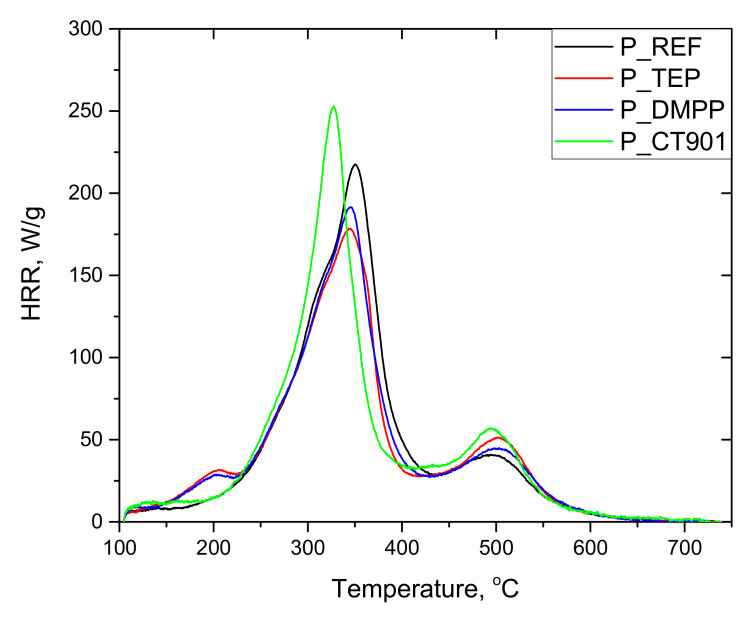
HRR curves of rigid polyurethane foams without the addition of bio-polyol.

**Figure 10:**
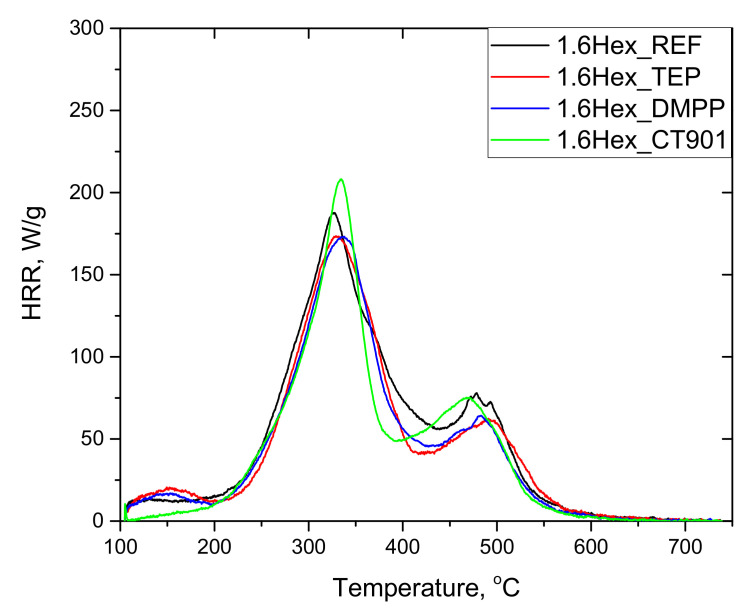
HRR curves of rigid polyurethane foams with 40 wt.% bio-polyol.

The tests of RPURFs using the pyrolysis combustion flow calorimeter (PCFC) determined the total heat released (THR), heat release capacity (HRC), and maximum heat release rates at decomposition stages (PHRR), as well as the time and temperature at which the HRR peak occurs. The obtained data are presented in Table 5. Based on the obtained results, it can be concluded that the thermal decomposition of the tested materials takes place mainly in two stages (Figure 9 and Figure 10). The first stage of degradation takes place at temperatures from 200 °C to 420 °C, and the second from 420 °C to 560 °C. The temperature range of the first stage corresponds to the decomposition of urethane bonds, urea bonds, and polyol segments into isocyanates, amines, aldehydes, ketones, carbon dioxide, and water, among others. The second stage corresponds to the decomposition of isocyanates and aromatic rings, as well as further degradation of the charred residue [14,39,40]. The peak HRR in foams with TEP and DMPP added at the temperature of about 180 °C may be the result of the presence of flame retardants in the gas phase, due to their low boiling points [14].

Each material modified with the flame retardant had a lower THR value compared to the foams without flame retardants. In the case of foams obtained exclusively from petrochemical polyol, the most significant reduction in THR was caused by the addition of DMPP, and for the foams containing the bio-polyol and Addforce CT 901.

The HRC parameter, which characterizes the material’s potential to release heat during combustion, is considered a key parameter in determining the reaction to fire of materials tested by PCFC [41]. The HRC value follows the same trend as the PHRR of the fastest stage due to the fact that HRC = PHRR/heating rate [41,42]. 1.6Hex_REF foam as well as other materials containing flame retardants and modified with the bio-polyol were characterized by lower HRC values compared to respective foams obtained from petrochemical polyol. The introduction of TEP and DMPP reduced HRC in both type systems, but for P_TEP and P_DMPP foams the modifiers made more significant differences than for 1.6Hex_TEP and 1.6Hex_DMPP foams compared to materials without any flame retardant. For foams with the addition of Addforce CT 901, the HRC value increased, which is the result of the decomposition of the flame retardant at temperatures close to the decomposition temperature of the material. This allows the gaseous decomposition products of the material to interact with the flame retardant, and thus effectively reducing the flammability by the use of the substance [6]. The same effect resulted in higher PHRR (1) values compared to unmodified foams.

The PHRR (2) value was higher for the foams with the addition of bio-polyol compared to the foams without the bio-polyol. Modified materials obtained only from petrochemical polyol were characterized by a higher PHRR (2) value compared to P_REF.

RPURFs with the addition of bio-polyol achieved PHRR (1) and PHRR (2) in a shorter time and at a lower temperature than foams obtained only from petrochemical polyol. This may be due to more flexible segments in the materials containing the bio-polyol. It follows that PUR bio-foams were less thermally stable.

The combustion test performed using a cone calorimeter allows to obtain data determining the behavior of the material during combustion, such as time to ignition (TTI), total heat release (THR), peak heat release rate (PHRR), average heat release rate (Av-HRR), maximum average rate of heat emission (MARHE), average effective heat of combustion (Av-EHC), total smoke release (TSR), total smoke production (TSP), average yield of CO (Av-COY), and average yield of CO_2_ (Av-CO_2_Y). These parameters make it possible to compare the flammability of different materials basing on the test with using much larger sample comparing to the PCFC test. However, the most important information on flammability is provided by HRR curve as a function of time [43].

**Figure 11:**
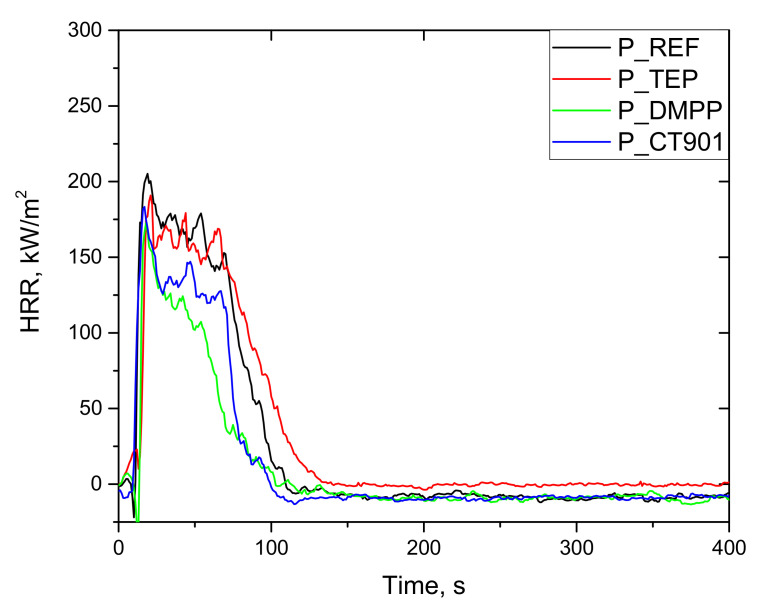
HRR curves (CONE) of rigid polyurethane foams without the addition of bio-polyol.

**Figure 12:**
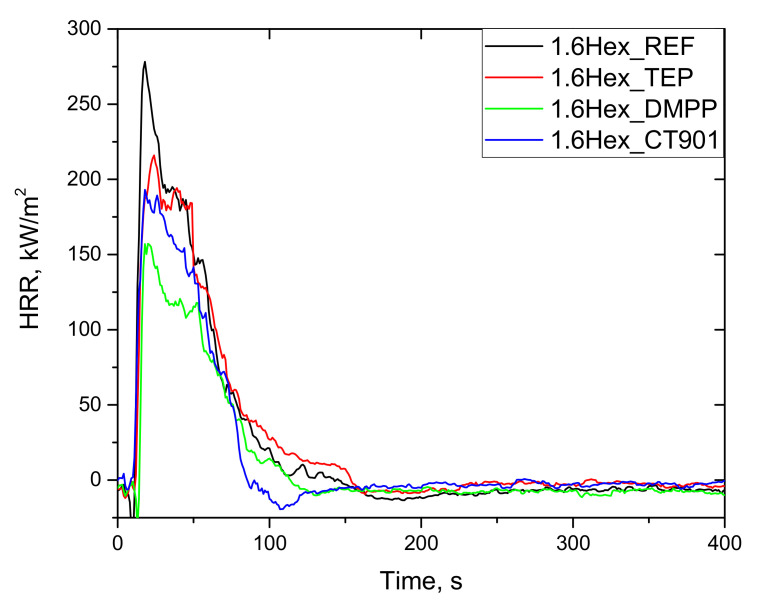
HRR curves (CONE) of rigid polyurethane foams with 40 wt.% bio-polyol.

As shown in Figure 11 and Figure 12, the heat release rate quickly increased immediately after ignition and reached a maximum peak. After reaching PHRR, the rate of heat release decreased as a result of char layer formation on the surface of foam as well as the effect of gas phase flame retardants. Detailed data are presented in Table 6. All foams obtained had a TTI of about 4 s. The addition of flame retardants reduced the PHRR value. This is particularly evident for bio-polyol modified materials, where the introduction of TEP, DMPP, and Addforce CT 901 lowered PHRR by 20%, 38%, and 33%, respectively, compared to 1.6Hex_REF foam. Despite the higher PHRR for 1.6Hex_REF foam comparing to P_REF foam, materials modified with the same flame retardant had a similar PHRR value in both systems, which is consistent with the results of the oxygen index test.

Interpretation of the data obtained after the cone calorimeter test can be performed using the MARHE value, which determines the risk of fire development [44]. All flame retardants in both systems reduced MARHE value, and the lowest value among the tested foams was for the P_DMPP foam and 1.6Hex_DMPP foam equaled 97 kW/m^2^ and 99 kW/m^2^, respectively, which is a reduction of 29% and 37% compared to their reference foams.

The total heat emission (THR) is also an important parameter for determining flammability of materials. The addition of TEP to both systems resulted in a slight reduction in THR as compared to the reference foams. The materials obtained, according to both systems containing DMPP and Addforce CT 901, had a lower THR value by 37% and 28%, respectively, compared to the unmodified foams.

The production of smoke and toxic products of combustion is as dangerous as fire. Therefore, the important parameters needed to fully describe the combustion process are TSR, TSP, CO_2_Y, and COY (Table 7). The introduction of phosphorus flame retardants led to an increase in TSP and TSR during combustion. This may be attributed to the fact that the flame retardants effectively stop the burning process by emitting incompletely burned fragments of PUR matrix [45]. Foams modified with each of flame retardants characterized by a higher COY and lower CO_2_Y, which results from incomplete combustion and leads to higher CO/CO_2_ ratio.

EHC describes the degree of combustion of volatile products resulting from the pyrolysis of the material [46]. All modified materials had lower EHC and residue after burning, as well as higher TSR and TSP value than the reference foams, which may be due to the fact that flame retardants act mainly in the gas phase [47]. The lower EHC value and higher smoke production result from the higher content of non-flammable decomposition products [44].

**Figure 13:**
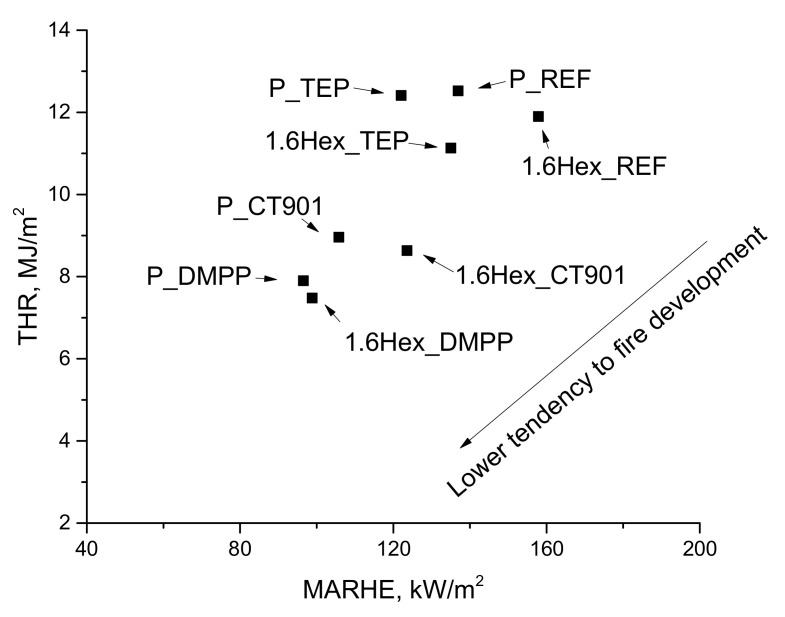
Dependence of the total heat release on MARHE.

**Figure 14:**
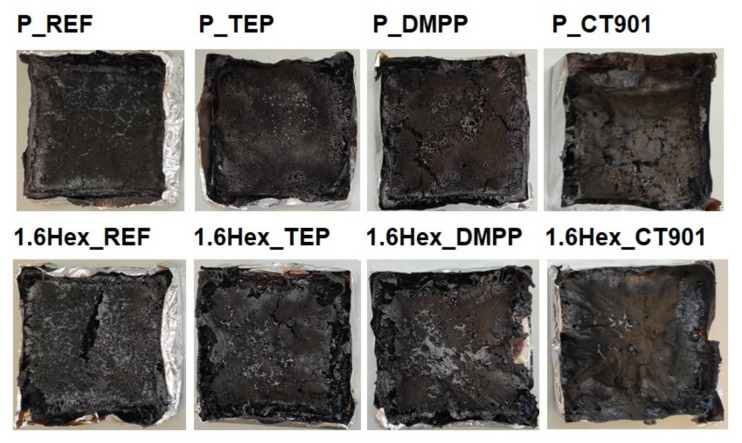
Photos of the samples after the cone calorimeter test.

The flammability of materials can also be determined by the dependence of the total heat released on the MARHE (Figure 13). Materials modified with flame retardants should have lower THR and MAHRE values compared to unmodified foams, which translates into a lower tendency to fire development [48]. In the analyzed foam materials, the tendency to fire development decreased the most after adding DMPP to both type of PUR systems, and the smallest change was shown by foams with TEP. Figure 14 shows the photos of the samples after the cone calorimeter test. Little cracked char residue remains after burning the obtaining foam materials. This shows little promotion of char formation of used flame retardants.

## 4. Conclusions

The introduction of 20 wt.% additive flame retardants to polyurethane systems in relation to the weight of polyol influenced the physical and mechanical properties of rigid polyurethane foams. The change in the reactivity of the systems, confirmed by the FOAMAT^®^ analysis, as well as the measurement of characteristic process times, disrupted the cellular structure, resulting in a decrease in the compressive strength of the foams, especially in the case of the addition of cyclic phosphonates. The low thermal conductivity of all obtained materials allows them to be used as thermal insulation. High hygroscopicity and good water solubility of cyclic phosphonates increased the water absorption of the P_CT901 and 1.6Hex_CT901 foams and increase the heat conduction coefficient under the influence of water or moisture.

P_DMPP and 1.6Hex_DMPP foams were characterized by the highest oxygen index in both systems, which may be due to the highest phosphorus content in the analyzed flame retardants. All modified materials had an oxygen index above 21 vol%; therefore, they can be classified as self-extinguishing materials [49]. In the pyrolysis combustion flow calorimeter test, all modified materials had a lower total heat release compared to unmodified materials. The modifications also reduced the heat release rate in the first stage of decomposition of polyurethane, except for foams with Addforce CT 901, due to the decomposition of flame retardant at a temperature close to the temperature of the first stage of polyurethane decomposition.

The applied flame retardant lowered the parameters obtained during the cone calorimeter test, such as THR, PHRR, Av-EHC, and MAHRE. These changes were more significant in foams with bio-polyol and containing DMPP or Addforce CT 901. The flame retardants increased TSR, TSP, and Av-COY, which is characteristic for flame retardants that act in gas phase where they can inhibit radical chain reaction. The analysis of obtained parameters showed that the modified rigid polyurethane foams have a lower tendency to fire development compared to the reference foams, which is particularly noticeable for materials with addition of DMPP.

## Figures and Tables

**Table 1:** Formulation of rigid polyurethane foams.

Foam Symbol	RF-551 Polyol, g	1.6Hex Bio-Polyol, g	Polycat^®^ 218 Catalyst, g	L-6915 Surfactant, g	Total Water, g	PMDI Isocyanate, g	Flame Retardant, g
P_REF	100	0	1.5	1.5	3.40	169.6	0
P_TEP							20
P_DMPP							20
P_CT901							20
1.6Hex_REF	60	40	1.5	1.5	3.23	148.0	0
1.6Hex_TEP							20
1.6Hex_DMPP							20
1.6Hex_CT901							20

**Table 2:** Parameters of foams cell structure in the direction parallel to the foam rise.

Foam Symbol	Number of Cells/1 mm^2^	Average Cross-Sectional Area of Cells, mm^2^ ·10^3^	Anisotropy Index
P_REF	40 ± 2	11.95 ± 0.89	1.23 ± 0.05
P_TEP	33 ± 3	12.22 ± 0.92	1.18 ± 0.03
P_DMPP	31 ± 2	15.29 ± 0.75	1.28 ± 0.06
P_CT901	44 ± 4	10.98 ± 1.04	1.28 ± 0.07
1.6Hex_REF	39 ± 2	12.89 ± 0.93	1.24 ± 0.05
1.6Hex_TEP	39 ± 2	10.82 ± 0.73	1.04 ± 0.04
1.6Hex_DMPP	34 ± 2	13.45 ± 0.64	1.10 ± 0.04
1.6Hex_CT901	57 ± 2	8.45 ± 0.84	1.18 ± 0.05

**Table 3:** Parameters of foams cell structure in the direction perpendicular to the foam rise.

Foam Symbol	Number of Cells/1 mm^2^	Average Cross-Sectional Area of Cells, mm^2^ ·10^3^	Anisotropy Index
P_REF	36 ± 2	14.19 ± 0.72	1.11 ± 0.03
P_TEP	40 ± 2	10.86 ± 0.84	1.07 ± 0.03
P_DMPP	38 ± 3	11.20 ± 0.85	1.00 ± 0.03
P_CT901	58 ± 2	8.20 ± 0.74	1.04 ± 0.04
1.6Hex_REF	50 ± 2	9.95 ± 0.86	1.04 ± 0.06
1.6Hex_TEP	49 ± 3	8.32 ± 0.76	0.88 ± 0.02
1.6Hex_DMPP	46 ± 2	9.02 ± 0.54	0.90 ± 0.03
1.6Hex_CT901	74 ± 2	5.98 ± 0.22	0.91 ± 0.02

**Table 4:** Selected physical and mechanical properties of RPURFs.

Foam Symbol	Apparent Density, kg/m^3^	Content of Closed Cells, %	Water Absorption, vol%	Conductivity Coefficient, mW/m·K	Brittleness, %	Compression Strength, kPa	
						Parallel	Perpendicular
P_REF	39.8 ± 0.3	92.3 ± 1.3	5.9 ± 0.6	24.55 ± 0.33	3.89 ± 0.35	221.7 ± 7.8	174.8 ± 4.9
P_TEP	41.9 ± 0.1	90.8 ± 2.0	4.4 ± 0.2	25.51 ± 0.24	5.04 ± 0.15	216.5 ± 7.9	184.8 ± 4.9
P_DMPP	40.3 ± 1.3	91.3 ± 1.8	5.3 ± 0.4	25.69 ± 0.40	5.40 ± 0.56	211.0 ± 5.6	148.3 ± 3.5
P_CT901	40.7 ± 1.0	93.5 ± 2.3	7.1 ± 0.9	25.15 ± 0.04	4.94 ± 0.12	241.0 ± 10.6	149.3 ± 6.2
1.6Hex_REF	38.3 ± 0.3	93.1 ± 0.6	5.8 ± 0.1	25.35 ± 0.21	3.29 ± 0.38	210.3 ± 1.0	143.8 ± 9.8
1.6Hex_TEP	41.6 ± 0.7	89.0 ± 2.0	5.6 ± 0.2	26.02 ± 0.17	4.28 ± 0.05	203.8 ± 5.9	153.5 ± 4.9
1.6Hex_DMPP	41.1 ± 1.4	89.1 ± 2.1	6.8 ± 0.1	25.78 ± 0.27	4.35 ± 0.54	186.8 ± 9.5	131.0 ± 6.7
1.6Hex_CT901	38.8 ± 0.4	91.2 ± 1.2	9.6 ± 0.5	25.32 ± 0.27	5.05 ± 0.17	187.8 ± 6.4	87.6 ± 7.0

**Table 5:** Test results for PUR foams on a pyrolysis combustion flow calorimeter.

Foam Symbol	THR, kJ/g	HRC, J/g·K	Temp. (1), °C ^a^	Time (1), s ^b^	PHRR (1), W/g ^c^	Temp. (2), °C ^d^	Time (2), s ^e^	PHRR (2), W/g ^f^
P_REF	27.0 ± 0.5	251 ± 9	343 ± 8	304 ± 6	225.6 ± 9.5	496 ± 2	454 ± 7	36.0 ± 2.6
P_TEP	24.9 ± 0.6	209 ± 10	347 ± 5	309 ± 9	188.8 ± 9.8	501 ± 1	460 ± 6	50.8 ± 2.9
P_DMPP	25.7 ± 0.3	232 ± 6	347 ± 5	306 ± 2	211.7 ± 5.7	491 ± 4	442 ± 4	45.1 ± 5.7
P_CT901	26.9 ± 0.5	281 ± 1	326 ± 1	289 ± 1	251.7 ± 0.9	495 ± 1	455 ± 1	56.3 ± 0.4
1.6Hex_REF	29.7 ± 0.3	206 ± 6	327 ± 4	283 ± 7	187.5 ± 5.0	478 ± 1	431 ± 6	71.8 ± 9.5
1.6Hex_TEP	27.3 ± 0.3	197 ± 9	332 ± 2	285 ± 6	185.6 ± 0.1	472 ± 6	441 ± 4	59.4 ± 3.6
1.6Hex_DMPP	26.6 ± 0.6	189 ± 4	332 ± 6	287 ± 9	170.9 ± 2.5	483 ± 2	436 ± 3	67.8 ± 5.8
1.6Hex_CT901	25.8 ± 0.9	236 ± 6	330 ± 5	290 ± 9	212.8 ± 8.0	489 ± 1	436 ± 8	80.6 ± 8.1

^a^—temperature for maximum heat release rate (stage 1). ^b^—time for maximum heat release rate (stage 1). ^c^—maximum heat release rate (stage 1). ^d^—temperature for maximum heat release rate (stage 2). ^e^—time for maximum heat release rate (stage 2). ^f^—maximum heat release rate (stage 2).

**Table 6:** The results of cone calorimeter tests.

Foam Symbol	TTI, s	THR, MJ/m^2^	PHRR, kW/m^2^	Av-HRR, kW/m^2^	MARHE, kW/m^2^	Av-EHC, MJ/kg
P_REF	5 ± 1	12.5 ± 0.7	206.5 ± 3.9	36.4 ± 0.6	137 ± 4	13.8 ± 0.8
P_TEP	5 ± 1	12.4 ± 0.5	203.3 ± 16.6	39.9 ± 3.7	122 ± 9	12.2 ± 0.5
P_DMPP	5 ± 1	7.9 ± 1.1	177.0 ± 6.3	20.4 ± 1.6	97 ± 4	7.3 ± 0.4
P_CT901	4 ± 1	9.0 ± 0.2	171.2 ± 14.3	24.9 ± 1.3	106 ± 9	8.2 ± 0.6
1.6Hex_REF	4 ± 1	11.9 ± 0.7	278.3 ± 16.8	35.3 ± 2.1	158 ± 3	13.6 ± 0.7
1.6Hex_TEP	4 ± 1	11.1 ± 1.8	222.0 ± 21.4	35.0 ± 7.1	135 ± 10	11.4 ± 0.7
1.6Hex_DMPP	4 ± 1	7.5 ± 0.4	173.2 ± 14.5	21.2 ± 1.6	99 ± 7	7.5 ± 0.4
1.6Hex_CT901	4 ± 1	8.6 ± 0.5	186.2 ± 9.6	25.4 ± 2.2	124 ± 8	8.4 ± 0.3

**Table 7:** Smoke emission behaviors and residue of polyurethane foam.

Foam Symbol	TSR, m^2^/m^2^	TSP, m^2^	Av-COY, kg/kg	Av-CO_2_Y, kg/kg	CO/CO_2_ Weight Ratio	Residue, %
P_REF	541 ± 44	4.78 ± 0.39	0.39 ± 0.02	3.46 ± 0.13	0.113 ± 0.006	22.2 ± 3.9
P_TEP	965 ± 43	8.53 ± 0.38	0.77 ± 0.06	2.68 ± 0.10	0.287 ± 0.027	14.6 ± 2.1
P_DMPP	796 ± 24	7.04 ± 0.21	0.86 ± 0.03	2.59 ± 0.10	0.332 ± 0.022	20.6 ± 2.0
P_CT901	894 ± 61	7.90 ± 0.54	0.76 ± 0.03	2.55 ± 0.08	0.300 ± 0.011	18.6 ± 1.6
1.6Hex_REF	547 ± 49	4.84 ± 0.44	0.40 ± 0.03	3.47 ± 0.12	0.116 ± 0.006	25.1 ± 1.4
1.6Hex_TEP	849 ± 48	7.51 ± 0.43	0.69 ± 0.04	2.85 ± 0.14	0.242 ± 0.006	18.2 ± 1.3
1.6Hex_DMPP	791 ± 39	6.99 ± 0.34	0.81 ± 0.02	2.46 ± 0.12	0.330 ± 0.008	22.7 ± 1.1
1.6Hex_CT901	811 ± 21	7.17 ± 0.19	0.73 ± 0.05	2.62 ± 0.12	0.279 ± 0.011	18.7 ± 2.4

## Data Availability

Data are contained within the article.
